# Systemic markers of inflammation are independently associated with S100B concentration: results of an observational study in subjects with acute ischaemic stroke

**DOI:** 10.1186/1742-2094-7-71

**Published:** 2010-10-29

**Authors:** Christopher Beer, David Blacker, Michael Bynevelt, Graeme J Hankey, Ian B Puddey

**Affiliations:** 1 Western Australian Centre for Health and Ageing, University of Western Australia; 2 School of Medicine and Pharmacology, University of Western Australia; 3 Centre for Medical Research, Western Australian Institute for Medical Research; 4 Royal Perth Hospital, Perth, Western Australia; 5 Sir Charles Gairdner Hospital, Perth, Western Australia

## Abstract

**Background:**

Vascular dysfunction and brain inflammation are thought to contribute to the pathophysiology of cerebral injury in acute stroke. However acute inflammation and vascular dysfunction may simply be markers of an acute phase response to cerebral injury, reflecting the size of the cerebral lesion. We aimed to determine if systemic markers of vascular dysfunction and inflammation are independently associated with concentrations of the astroglial protein S100B, a marker of brain injury, in participants with acute ischaemic stroke.

**Methods:**

Fifty-seven men and women recruited within 96 hours of acute ischaemic stroke at two tertiary hospitals participated in this cross sectional observational study. Clinical, imaging (stroke lesions area measured with perfusion CT) and laboratory data were the independent variables and co-variates. The outcome variable was serum S100B concentration, analysed by multivariate regression.

**Results:**

High sensitivity-CRP (*B *= 0.41) and lesion area (*B *= 0.69) were independently associated with S100B concentration (R^2 ^= 0.75, p < 0.01). Other variables with significant univariate associations with S100B concentration were not independently associated with S100B concentration in the final multivariate model.

**Conclusion:**

The degree of systemic inflammation is associated with S100B concentration in acute ischaemic stroke, independent of the size of the ischaemic lesion.

## Background

Acute ischaemic stroke is associated with a rise in systemic markers of endothelial activation, inflammation and oxidative stress [1-6]. At the site of brain injury vascular dysfunction, oxidative stress and brain inflammation are thought to contribute to the pathophysiology of cerebral injury in acute stroke [2,7]. However, it is uncertain whether these factors simply represent an "acute phase" response to the cerebral injury, and associated complications such as immobility, or are important independent predictors of the degree of cerebral injury resulting from an acute ischaemic insult.

Much evidence shows that inflammation in the setting of acute ischaemic stroke is associated with infarct size, supporting the hypothesis that inflammation in acute stroke primarily reflects an acute phase response determined by the degree of cerebral injury [1,8]. However the magnitude of the acute phase response appears to also be independent an predictor of clinical outcome [9]. In patients with lacunar syndromes, who tend to have small volume lesions, progression of neurologic symptoms is associated with markers of the acute phase response [10]. These data suggest that inflammation may be an important independent factor in the pathophysiology of acute ischaemic stroke.

S100B is a peptide derived largely from astrocytes. In normal physiology S100B has multiple local regulatory effects on cell division and metabolism. Ischaemia is associated with raised S100B levels [11,12]. This is thought to be due to damage to astrocytes. Thus S100B concentrations are marker of the degree and severity of cellular injury in acute ischaemic stroke.

We conducted this study to test the hypothesis that endothelial function, inflammation and oxidative stress are independently associated with the degree of cellular injury in acute ischaemic stroke. We aimed to determine if endothelial function, inflammation and oxidative stress are associated with S100B concentration in acute ischaemic stroke, and in particular if any association is independent of infarct size.

## Methods

### Design

Cross-sectional observational study.

### Setting and participants

Patients were recruited at two teaching hospitals in Perth, Western Australia between May 2005 and November 2008. Patients admitted with acute ischaemic stroke within 96 hours of onset were eligible to participate. Exclusion criteria were: blood glucose level > 13 mmol/L; acute co-morbid condition; creatinine >120 umol/L; haemorrhage seen on initial CT; and history of sensitivity to contrast. Clinical records were reviewed subsequent to the patient's discharge to confirm a final clinical diagnosis of an acute cerebral ischaemic event and classify the clinical syndrome using the Oxfordshire Community Stroke Project classification [13].

### Assessments

Clinical characteristics were assessed using the National Institutes of Health Stroke Scale (NIHSS),[14] Modified Barthel Index (MBI) [15] and Modified Rankin Scale (MRS) [16]. Laboratory data were collected to assess S100B concentrations [11], inflammation (C-reactive protein [CRP] and fibrinogen [9]), endothelial activation (E-selectin [1]), endothelial cell damage (Von Willebrand factor [vWF] [1]) and oxidative stress [F2-isoprostanes] [4]). With the exception of F2-isoprostanes, all assays were performed by the PathWest Laboratory Medicine Units at Royal Perth and Sir Charles Gairdner Hospitals, using routine collection and analysis procedures. For analysis of F2-isporostanes, 5 ml of whole venous blood was collected into cold EDTA tubes containing reduced glutathione and centrifuged as soon as possible at 1000 g for 10 min at 4°C. The plasma was protected from oxidation by the addition of butylated hydroxytoluene at a final concentration of 20 μg/ml plasma and stored at -80°C until analysis by gas chromatography/mass spectrometry [17]. Blood pressure was assessed using validated ^,^[18,19] oscillometric ambulatory blood pressure monitors (Oscar 2, SunTech Medical, Morrisville NC USA) worn by participants for 24 hours after enrolment.

Participants underwent perfusion CT scanning. Five patients were imaged prior to July 2005. A single 10 mm slice was taken at the level of the third ventricle. Cycle time was 1 s. Subsequent to July 2005 8 slices were acquired on mulitdetector machines (Philips Brilliance 64 slice scanner). 40 mL of non-ionic contrast ('Optiray') was administered over 10 seconds using an intravenous cannula in the antecubital fossa. The multidetector machine protocol was: length 40 mm, thickness 5 mm, increment 0 mm, kV 120, mAs 100, cycle time 1.5, cycles 45, Resolution High, Collimation 32 × 1.25, Rotation time 0.4 sec, Filter Smooth UA, matrix 512. Post processing and analysis was completed on Philips and GE workstations. When multiple slices were available, the slice with greatest lesion area evident on the plain CT was chosen for analysis. Infarct size was measured by an experienced neuroradiologist (MB) blinded to treatment allocation.

### Data analysis

SPSS for MS Windows was used to analyse data. Log-normal data were log transformed prior to analysis. Non-normal data were analysed using non parametric tests. Categorical variables were assessed with the chi squares or fisher exact test. Linear regression modeling was used to examine the association between predictor variables and concentration of S100B. Only non-missing data were included in the modeling exercise. This commenced with a series of univariate models to identify all candidate predictor variables. These significant variables were then included in a multivariate logistic regression model and removed using a backward stepwise process if their significance was not retained. Box-plots of the remaining variables were re-inspected to ensure that outlying values were not present, before producing the final parsimonious model.

### Ethical considerations

The study was approved by the Royal Perth and Sir Charles Gairdner Hospitals Ethics Committees. Able participants provided written informed consent. If there was uncertainty regarding the person's ability to provide informed consent agreement to study participation was also sought from the person's next of kin. The procedures followed were in accordance with the Helsinki Declaration.

## Results

### Cohort characteristics

An heterogeneous group of 57 acute ischaemic stroke patients participated in the study (Table 1). The mean age of participants was 68 years. Participants were enrolled a mean of 53 hours after the onset of symptoms. Although there was wide variability in the sample, many participants had moderate - severe stroke (the mean NIH stroke scale score was 10, 40% of participants had a modified Rankin score of 4 or 5 and the mean lesion area was 10 cm^2^).

**Table 1:** Cohort characteristics

Variable	Mean ± SD or n(%)
Age (years)	67.8+13.7

Male (%)	39 (68%)

Enrolled (hours)	52.6 ± 21.6

Hypertension	35 (61%)

Atrial fibrillation	10 (18%)

Hypercholesterolaemia	11 (19%)

Diabetes	13 (23%)

Smoking	21 (37%)

Aetiology	
Large vessel	6 (11%)
Small vessel	8 (14%)
Cardioembolic	24 (42%)
Other	1 (2%)
Unknown	18 (32%)

Syndrome	
Total anterior circulation	14 (25%)
Partial anterior circulation	26 (46%)
Lacunar	11 (19%)
Posterior circulation	6 (11%)

Systolic blood pressure (mmHg)	142.1 ± 19.3

Diastolic blood pressure (mmHg)	80.2 ± 11.2

NIH stroke scale score	8.5 ± 7.8

Modified Rankin scale score	
0-3	17 (30%)
4-5	40 (70%)

Modified Barthel index score	39.3 ± 33.9

Glucose (mmol/L)	6.3 ± 1.6

Homocysteine (umol/L)	9.9 ± 3.8

High sensitivity-CRP (mg/L)	15.7 ± 27.9

Fibrinogen (g/L)	4.2 ± 1.2

E-selectin (ng/mL)	30.5 ± 34.0

von Willebrand Factor (%)	158.7 ± 61.6

S100B (ug/L)	0.5 ± 0.8

F2-Isoprostanes (nmol/L)	3.2 ± 4.2

Lesion area (mm^2^)	1001.9 ± 1359.8

### Factors associated with S100B concentrations

Significant univariate associations were found between age, stroke syndrome, neurologic impairment (NIHSS Score), disability (Modified Barthel Index score), lesion area, hs-CRP and serum concentration of S100B. These are shown in Table 2. However, of these factors, only hs-CRP (*B *= 0.41) and lesion area (*B *= 0.69) remained independently associated with S100B concentration in the final parsimonious multivariate model. Results of this model are also listed in Table 2. The multivariate model, including 54 participants with no missing data, showed that hs-CRP and lesion area explained the majority of variance in S100B (Adjusted R^2 ^= 0.76, p < 0.01).

**Table 2:** Univariate and multivariate relationships (S100B as dependent variable) analysed by linear regression

Predictor	B	SE B	*B*	p
**Univariate Relationships**				

Age	0.01	0.01	0.27	0.05

Gender	-0.10	0.18	-0.07	0.60

Hours to enrolment	0.00	0.00	0.07	0.62

Hypertension	-0.06	0.17	-0.05	0.73

Atrial fibrillation	0.19	0.22	0.12	0.40

Hypercholesterolaemia	-0.20	0.22	-0.12	0.37

Diabetes	0.02	0.20	0.02	0.90

Smoking history	-0.09	0.17	-0.07	0.60

Aetiology	-0.03	0.06	-0.06	0.66

Clinical syndrome	-0.43	0.08	-0.61	0.00

Systolic blood pressure	0.00	0.00	0.00	0.99

Diastolic blood pressure	0.00	0.01	-0.07	0.58

NIHSS score	0.56	0.20	0.36	0.01

Modified Rankin	0.00	0.00	1.10	0.28

Modified Barthel	-0.01	0.00	-0.36	0.01

Glucose	1.52	0.84	0.25	0.08

Homocysteine	0.01	0.52	0.00	0.99

Hs-CRP	0.56	0.12	0.55	0.00

Fibrinogen	0.10	0.07	0.20	0.16

E-Selectin	-0.09	0.29	-0.04	0.75

von Willebrand factor activity	0.00	0.00	2.26	0.03

F2-Isoprostanes	0.04	0.31	0.02	0.89

Lesion area	0.73	0.08	0.77	0.00

**Final multivariate model**				

Hs-CRP	0.42	0.07	0.41	0.00

Lesion area	0.65	0.07	0.69	0.00

## Discussion

### Main findings

These data show that the relationship between S100B concentration and inflammation in participants with acute ischaemic stroke is independent of infarct size. These data establish an independent association between markers of inflammation and glial injury, suggesting that the inflammatory response to acute ischaemic injury may be a therapeutic target. The concept that limiting inflammation may limit brain injury is supported by evidence that components of the acute phase response to cerebral injury are deleterious. For example, CRP is known to inhibit generation of endothelial progenitor cells, which are necessary for angiogensis [20]. Future intervention studies are required to determine whether modifying the inflammatory response may limit cellular injury in acute ischaemic stroke.

### Results in context of other studies

S100B has previously been found to be correlated with CRP in subjects with acute ischaemic stoke at 12, 24 and 72 hours after stroke, after controlling for gender, age and leukocyte count [21]. The present results extend those findings to show that the association between CRP and S100B is also independent of infarct size and a range of other potentially confounding variables.

Since our study was planned there has been an accumulation of data regarding the potential importance of inflammation and S100B concentrations in subjects with acute ischaemic stroke and other neurologic disorders. Both CRP concentrations at 72 hours, and S100B concentrations at twelve hours, have been found to be independently associated with clinical outcome following acute ischaemic stroke [22]. At nano-molar levels S100B has trophic effects which may an adaptive response to brain injury. However S100B at micromolar concentrations, may itself have neurotoxic (apoptotic) effects[23]. Over-expression of S100B has now been documented in a range of brain diseases [23]. Thus, although we were interested in S100B as a surrogate marker of clinical outcome, the higher concentrations of S100B may potentially cause, rather than simply reflecting, the degree of brain injury.

S100B also has pro-inflammatory effects. Although S100B is thought to be brain-specific, raised concentrations of S100B have been observed in systemic diseases without apparent neurologic involvement. Among subjects with systemic lupus erythematosus, concentrations of S100B were elevated (relative to controls) among subjects without central nervous system involvement, although to a lesser extent than among subjects with neurologic or psychiatric manifestations [24]. This could be because S100B is a sensitive marker of sub-clinical neurologic disease. It has since been postulated that S100B may act as an inflammatory mediator in subjects with non-neurologic disease. However S100B was found not to be associated with CRP in renal transplant recipients [25].

### Strengths of our study

Ours is one of few studies to simultaneously assess infarct size, clinical variables and systemic markers of inflammation, oxidative stress and vascular function. We used a conservative approach in the modeling exercise. We included both sensitive clinical scales and imaging measures. For example, the NIHSS correlates with infarct volume (r = 0.68) and is a strong predictor of outcome after stroke [26,27]. Thus substantial residual confounding of the observed association between inflammation and S100B concentrations by lesion severity seems unlikely. We also accounted for the heterogeneity of acute ischaemic stroke syndromes by assessing stroke aetiology and clinical sub-type.

### Limitations

There are several limitations to the study. We did not assess for other potential causes of inflammation (such as deep venous thrombosis and pulmonary atelectasis) which may confound the association between neurologic lesions and inflammation. Other potential confounding variables which were not controlled for include the degree of leukoariosis, and medical therapies. Determining lesion volume (rather than lesion size in one plane) would also have allowed greater confidence that differences due to lesion severity were adequately controlled in the multivariate models.

Interpretation of our results is also limited as S100B was only measured once. We were thus unable to account for differences in inflammatory status prior to the acute stroke event. The cross sectional design prevents any causal inferences being made. In addition, elevated S100B concentrations are not specific to ischaemic brain injury and the pattern of change in S100B levels is variable depending on the stroke subtype. Overall, serum S100B levels peak 2-3 days after an ischaemic stroke [12]. However in large middle cerebral artery (MCA) territory strokes serum S100B levels may not peak until 7 days [11]. Despite this variability, single S100B concentrations measured 2-3 days after non-lacunar ischaemic stroke were found to provide optimal predictive value compared to more complex measures [28]. Although S100B consistently appears to predict outcomes, data from subjects with traumatic brain injury indicate that the relationship between inflammation and alternative markers of brain injury (such as neuron specific enolase) may also warrant examination in subjects with acute ischaemic stroke [29].

## Conclusion

The degree of systemic inflammation is independently associated with S100B concentrations in subjects with acute ischaemic stroke.

## Competing interests

The authors declare that they have no competing interests.

## Authors' contributions

CB designed the study, carried out or supervised subject recruitment and collection of data, analysed the data and wrote the manuscript. DB assisted recruitment and data collection at the Sir Charles Gairdner site and assisted data interpretation and manuscript revision. MB analysed perfusion CT scans and assisted data interpretation and chapter revision. IBP and GH assisted in study design, data interpretation and manuscript revision. All authors have read and approved the final version of the manuscript.
